# Reformulation of the protein databank for real-time search of geometrical attributes of protein structures

**DOI:** 10.3389/fmolb.2025.1694750

**Published:** 2026-01-13

**Authors:** Musa Azeem, Christopher Lee, Aaron Hein, Christopher Ott, Homayoun Valafar

**Affiliations:** Molinaroli College of Engineering and Computing, University of South Carolina, Columbia, SC, United States

**Keywords:** dihedral angles, protein data bank, protein structure validation, ramachandran plot, sequence-structure relationship, structural bioinformatics

## Abstract

**Introduction:**

In this study, we introduce the design and implementation of PDBMine, a large-scale, queryable platform for mining sequence-structure statistics from the Protein Data Bank (PDB). PDBMine enables rapid analysis of local conformational trends across proteins by extracting dihedral angles and sequence patterns at scale. In addition to the design and implementation of PDBMine, we also present results validating its ability to return structurally meaningful information.

**Methods:**

We first assess the accuracy of its dihedral angle distributions by comparing them to established Ramachandran space and verifying expected behaviors of residues such as glycine and proline. We then use PDBMine to analyze the statistical properties of amino acid subsequences of length k = 1 to 5.

**Results:**

Our findings reveal that longer k -mers exhibit significant departures from statistical independence, suggesting context-dependent constraints on amino acid co-occurrence. We also show that increasing local sequence context restricts dihedral angle variability, with longer k -mers producing distributions that more closely align with experimentally observed backbone geometries. Finally, we present a high-dimensional clustering method for grouping full-sequence dihedral conformations, enabling identification of dominant local structural motifs.

**Discussion:**

These results highlight PDBMine’s potential as a versatile tool for structure validation, statistical modeling, and probing the principles that govern sequence-structure compatibility in proteins.

## 1 Introduction

Over the past 3 decades, significant advancements have been made in the field of protein structure determination, driven by both experimental and computational approaches (Kühlbrandt, 2014; Senior et al., 2020). Since the completion of the Human Genome Project (Collins et al., 2003), the demand for rapid and cost-effective structural elucidation of proteins has intensified, leading to the establishment of large-scale initiatives such as the Structural Genomics Initiative and the Protein Structure Initiative. These efforts aimed to accelerate the discovery of protein structures and expand our understanding of protein function, interactions, and evolution. However, experimental techniques such as X-Ray crystallography, nuclear magnetic resonance (NMR) spectroscopy, and cryo-electron microscopy (cryo-EM) remain time-intensive and resource-demanding (Bax and Clore, 2019; Bai et al., 2015), necessitating the development of computational structure prediction methods as an alternative.

To advance computational modeling, initiatives such as the Critical Assessment of Structure Prediction (CASP) (Moult et al., 1995) were established, providing a rigorous benchmarking framework for evaluating protein structure prediction algorithms. Early computational approaches, such as homology modelling (Marti-Renom et al., 2000), leveraged evolutionary relationships between proteins to infer structural information. More recently, deep learning-based models, exemplified by AlphaFold2 (Jumper et al., 2021), have demonstrated remarkable accuracy in predicting protein structures directly from their primary sequences, revolutionizing the field of structural bioinformatics. These computational breakthroughs heavily depend on data mining and knowledge extraction from the Protein Data Bank (PDB) (Berman et al., 2000), which serves as the primary repository of experimentally determined structures.

However, despite its indispensable role in structure prediction, the PDB remains limited for large-scale, data-driven analyses. Originally conceived as an archival system for individual structures rather than for geometric pattern mining (Kleywegt and Jones, 1998), it requires extensive post-processing to extract features such as torsion angles or residue-level relationships. This limits its direct use in machine learning workflows that rely on structured, readily accessible geometric data (Shortle, 1999).

To overcome these limitations, we introduced PDBMine (Cole et al., 2019), a reformulation of the PDB designed for efficient large-scale structural analysis. PDBMine provides direct access to geometrically relevant structural attributes such as dihedral angle and omega angles, burial distances of residues, and water accessibility; enabling rapid query-based examination of sequence-structure relationships. In this report, we demonstrate the validity of PDBMine by comparing its results for Ramachandran restraints to the traditionally accepted structural restraints (Lovell et al., 2003), ensuring consistency with traditionally accepted structural distributions (Lovell et al., 2003). Additionally, we extend conventional structural analysis by introducing higher-dimensional Ramachandran data, providing previously unexplored insights into backbone conformations. Beyond validation, we illustrate PDBMine’s practical applications through a series of case studies that highlight its uses in structural bioinformatics, protein design, and predictive modeling. By expanding the scope of structural analysis to multi-residue dihedral distributions, PDBMine offers a new paradigm for understanding local protein structure formation behavior.

## 2 Materials and methods

### 2.1 Overview of PDB

Established in 1971, the Protein Data Bank (PDB) (Berman et al., 2000), is the world’s primary repository for three-dimensional structural data of biological macromolecules. Currently, PDB archives 238,089 protein structures (statistics obtained on 20 June 2025), it is an indispensable resource for structural and computational biologists, enabling advancements in drug discovery, protein engineering, and molecular dynamic simulations.

The PDB is well regarded for its archives, serving as a comprehensive repository of biomolecular structures with rich metadata that includes items such as the atomic coordinates, experimental methods, and structural resolutions among other important information. It supports a range of structural determination techniques as well, such as X-ray crystallography, nuclear magnetic resonance spectroscopy, and cryo-electron microscopy, ensuring a broad coverage of biomolecular diversity. All of this data is freely available and universally accessible, its intuitive search tools enable users to filter data by sequence or structure to facilitate efficient exploration of the database.

Despite its extensive data repository, the PDB’s original archival design limits its use for modern large-scale data mining. Its organizational framework does not inherently support complex queries for structural parameters such as dihedral angles or residue level spatial constraints, features essential for advanced structural analysis.

The advent of artificial intelligence (AI) and machine learning (ML) has brought transformative potential to structural biology. We have seen the predictive and generative modeling power of applications such as AlphaFold2, which requires large, well-organized datasets. While such predictive frameworks have revolutionized the field, their accuracy and reliability remain fundamentally grounded in experimentally determined structures. The training, validation, and benchmarking of models like AlphaFold2 and RoseTTAFold (Baek et al., 2021) depend directly on empirical data curated within the Protein Data Bank (PDB), making the continued accessibility and reformulation of experimental information critical for sustaining progress in predictive modeling. However, in its current state, PDB’s structure presents several challenges for development of AI/ML applications on its own. For AI-driven approaches, its success often depends on a dataset that enables systematic mining of patterns. And for machine learning models, they often rely on structured and interpretable input data. While the PDB stores Cartesian atomic coordinates, these coordinates are high-dimensional and often redundant for pattern recognition tasks. Dihedral angles provide a lower-dimensional and biologically interpretable representation of local backbone conformation, and are therefore a natural feature space for machine learning and predictive modeling.

Thus, while the PDB remains a vital resource for structural biology, its focus on archival functions limits its applicability in contemporary computational research. Addressing these limitations is crucial to harnessing the full potential of AI and ML technologies, and enhancing access to systematically organized datasets will be increasingly important as these fields evolve.

### 2.2 Overview of PDBMine

PDBMine was developed to address these limitations by providing a more efficient framework for large-scale structural data mining (Cole et al., 2019). It reformulates atomic coordinate data into an analytically tractable representation by systematically extracting backbone dihedral angles ( ϕ , ψ , ω ) using the DSSP algorithm (Kabsch and Sander, 1983).

As noted earlier, PDB’s archiving of structural data typically falls into Cartesian representation of the atomic coordinates (X, Y, Z coordinates); these values are less intuitive for understanding local conformations, folding patterns, or secondary structure prediction. Instead, dihedral space representation ( ϕ , ψ , ω angles) can offer more meaningful representation of protein backbone conformations in aid of analyzing protein structure and dynamics. These angles define the torsional freedom of amino acids, effectively summarizing local structural geometry; whereas Cartesian coordinates would require complex calculations just to infer conformational patterns. PDBMine enables direct access to this higher-level representation, streamlining analysis that would otherwise require extensive preprocessing.

Additionally, PDBMine extends Ramachandran-based restraints beyond just a single amino acid by including contextual sequence information. For instance, while it is well demonstrated that glycine exhibits broader dihedral flexibility than other amino acids, the influence of neighboring residues on its conformational preferences remains poorly understood (Lakshmi et al., 2014). PDBMine enables such analysis by grouping dihedral distributions across multi-residue motifs, including pairs (e.g., Gly-Pro), triplets (e.g., Gly-Pro-Gly), and longer k-mers. This approach allows for a richer characterization of local structural tendencies, capturing how sequence context modulates backbone geometry.

By systematically indexing these multi-residue dihedral spaces, PDBMine provides data-driven insights that can be used to guide protein modeling, constrain predictions of viable backbone conformations, and reduce the computational complexity associated with protein structure determination. The ability to mine dihedral distributions in the context of various k-mer sequences, allows PDBMine to provide statistically derived structural constraints. This information can be integrated into computational approaches for structure prediction, molecular dynamic simulations, and validation of machine learning models aimed at protein folding.

PDBMine was originally introduced in 2019 as a reformulated database of protein structural information optimized for dihedral angle analysis (Cole et al., 2019). Since then, substantial improvements have been made to the underlying infrastructure to enhance usability, scalability, and performance. In particular, a major update in 2021 re-engineered the platform to support RESTful web services, containerized deployment, and high-speed queries.

### 2.3 Software implementation aspects

#### 2.3.1 Data preparation and processing

To construct a comprehensive local dataset of backbone conformations, we systematically downloaded all protein structures from the PDB. For each protein chain or model, backbone dihedral angles ( ϕ and ψ ) were computed using DSSP (Kabsch and Sander, 1983), which remains the standard algorithm for secondary-structure assignment and torsional geometry calculation. We note that an updated version has recently been released (Hekkelman et al., 2025), expanding support for newer structure formats, although the present work used the original implementation. The full description of the technical details on the creation of the PDBMine data has been previously reported (Cole et al., 2019). The resulting angular measurements were extracted at the residue level and aligned with the corresponding amino acid sequence to capture the structural context of each residue within the full-length protein. This allows the construction of an indexed database of sequence fragments with associated dihedral angle information. By performing these calculations in advance, we shifted the computational burden to the data preparation stage, thereby enabling faster response times during the downstream user queries.

#### 2.3.2 Sequence window search and indexing

**FIGURE 1:**
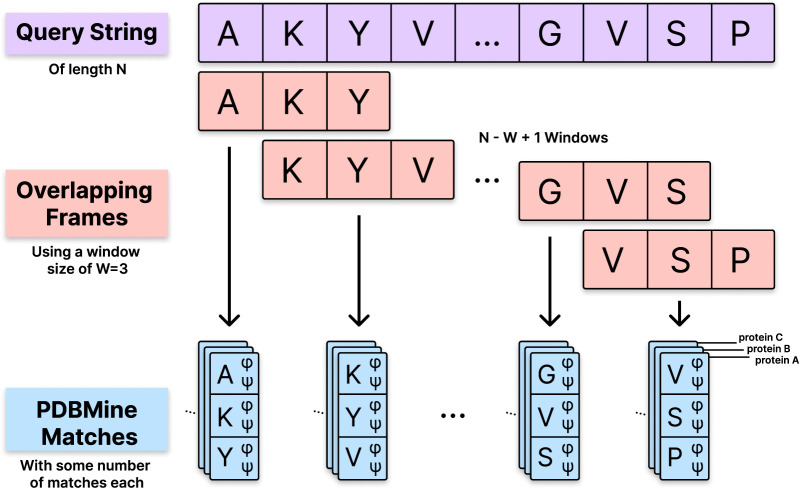
*Sequence windowing and dihedral angle retrieval in PDBMine*. A query sequence of length N is divided into overlapping windows of length W , producing N−W+1 windows. Each window is used to retrieve all matching fragments in the PDB that share the same amino acid sequence, along with their associated ϕ and ψ dihedral angles.

PDBMine employs a window-based indexing strategy. When a user submits an amino acid sequence of length N , along with a specified window size W , the query is partitioned into N−W+1 overlapping windows, each comprising W consecutive residues. The window of size W is shifted by one residue across the entire protein sequence. As illustrated in Figure 1, each window is then independently searched against the database to identify all matching sequence fragments across the PDB. For each match, the system returns the corresponding dihedral angles ( ϕ and ψ ) for the residues in the window. Because the same k -mer sequence may appear in multiple structural contexts (e.g., helices, strands, loops), the resulting dataset enables comparisons of how identical sequences adopt different backbone conformations in different proteins.

#### 2.3.3 User interface and usability

PDBMine is distributed via a Docker container, which enables users to run the application locally without needing a dedicated server. This version supports the same interactive query capabilities, including the ability to submit amino acid sequences in either one-letter (e.g., E Y V) or three-letter (e.g., Glu Tyr Val) codes and to define custom window sizes. The system processes each query and returns downloadable CSV files containing the matched k -mer fragments, the corresponding proteins, and their associated dihedral angles. In addition to the dihedral information reported for each of the k -mer windows, PDBMine also aggregates the torsion angles for each residue by aggregating the data for every instance of the shifting window in which the given residue appears. Therefore, the first and last k−1 residues of the sequence appear in 1 through k−1 windows, respectively. All other residues will appear in k windows (the first through the *k*th place in the window).

Docker-based distribution simplifies deployment and ensures that PDBMine can be executed in a consistent, reproducible environment across operating systems. The platform is designed to support a wide range of structural biology applications, allowing users to perform reproducible and large-scale structural analyses without dedicated infrastructure. To complement the containerized distribution, we have also restored a publicly accessible instance of the current PDBMine backend at https://ifestos.cse.sc.edu/PDBMine, providing users with a convenient browser based interface for testing and exploratory queries without requiring a local build.

#### 2.3.4 Performance and deployment

The computational performance of PDBMine depends primarily on the user’s available resources and deployment configuration. To assess system performance, we deployed PDBMine on an AWS t3.2xlarge instance (8 vCPUs, 32 GB RAM) and conducted several benchmark tests. A query on a 25-residue sequence with a 7-mer window completed in approximately 1 s, while the same sequence queried with a 2-mer window completed in 18 s. To further evaluate scalability, 100 proteins of varying lengths were selected at random from the PDB and queried sequentially with the same window size. The results remained consistent across sequence lengths, indicating that query time did not degrade with increasing input size.

The preprocessing step required to construct the full database (i.e., generating DSSP files and residue–position sets) took approximately 27 h on the same instance. Although precalculated DSSP annotations are publicly available, we chose to generate them locally to ensure consistent formatting with our parser and to avoid potential disruptions from future changes to external file formats or access methods. This design also enables seamless incremental updates, as only newly added or modified PDB entries require reprocessing. As such, this is a one-time cost incurred only during initial database construction or subsequent updates.

The complete Docker configuration, source code, and RESTful API framework are publicly available at https://github.com/ValafarLab/PDBMine. This containerized deployment allows users to reproduce performance benchmarks locally or on cloud infrastructure (e.g., AWS) and to access PDBMine’s functionality through its documented REST endpoints. The modular architecture ensures reproducible results while maintaining flexibility for users with different computational configurations.

As PDBMine continues to evolve, future updates will focus on integrating more advanced analytical tools and machine-learning models to enhance predictive insights into protein structure and function.

## 3 Results

In this section we explore various experiments to validate that PDBMine is functioning as intended and to demonstrate its utility in structural biology. We first validate the results of PDBMine by comparing the dihedral angles reported by PDBMine to the expected Ramachandran restraints. We then explore the abundance of different amino acids and their combinations in the PDB. Both of these exercises will serve to validate the accuracy of PDBMine by comparing its results to established structural benchmarks. Finally, we demonstrate the utility of PDBMine in a number of applications including structural motif discovery by examining the dihedral angle distributions of specific amino acid sequences and their surrounding residues.

Throughout the following sections, PDBMine is queried using the standard user interface method described in Section 2.3. Although the same PDBMine application is used throughout this work, we format our queries to PDBMine using two different methods. Whenever we query PDBMine with the entire amino acid sequence of a protein, we choose a window size and PDBMine applies the shifting window method as illustrated in Figure 1 to query the PDB with each window. This is referred to as Method 1 of querying PDBMine. Whenever we query PDBMine with a short sequence of amino acids, on the other hand, we specify a window size equal to the length of the subsequence. This way, PDBMine’s standard shifting window does not apply and the method only forms a single window. This method is used when we are only interested in the dihedral angles of a short subsequence of amino acids (4 residues, for example), rather than the entire sequence of a protein. This is referred to as Method 2 of querying PDBMine, and is summarized along with Method 1 as follows:

- Method 1 Query: PDBMine is queried with a full protein sequence of N amino acids and a window size of W , resulting in N−W+1 windows. Matches are found for each window, and the dihedral angles of the residues in each are returned, resulting in N−W+1 sets of W ( ϕ , ψ ) angle pairs.
- Method 2 Query: PDBMine is queried with a short subsequence of N amino acids and a window size of W=N , resulting in one window. Matches are found for the subsequence, and the dihedral angles of the residues in the subsequence are returned, resulting in one set of N ( ϕ , ψ ) pairs.

### 3.1 Validation of PDBMine results

ϕ and ψ dihedral angles in proteins adhere to the restricted space of the Ramachandran plot (Ramachandran et al., 1963; Kleywegt and Jones, 1996) (or R-space). The results of a PDBMine query, containing dihedral angles from proteins across the PDB, must adhere to this R-space (Lovell et al., 2003). As a first step in assessing the validity of PDBMine, we will compare the R-space created by PDBMine to traditional Ramachandran space distributions. In addition, we evaluated whether the dihedral angles returned by PDBMine for the amino acids glycine and proline exhibit their well-characterized conformational behaviors. Specifically, glycine is expected to form five distinct conformational clusters and proline should show a restricted angle at approximately ϕ≈−65° (Ho and Brasseur, 2005). To confirm the validity of PDBMine, we performed a series of PDBMine queries and analyses with Method 1 using the amino acid sequences of proteins from the PDB. We selected 5 proteins (PDB IDs: 4JSE, 7KVK, 4F0Z, 3IHU, and 7JHK) with an average sequence length of 375±87 residues (Jing et al., 2013; Samuels and Sevrioukova, 2021; Grigoriu et al., 2013; Joint Center for Structural Genomics, 2009; Hao et al., 2021). For each protein, a window size of 5, 6, or 7 was chosen at random. These window sizes were chosen to yield a significant, but not overwhelming, number of matches for each window. For each protein, we collected the dihedral angles of the residues across each window’s matches, resulting in approximately 3 million queried angle pairs. We expect these representative results of PDBMine to adhere to the same R-space as the dihedral angles found in models directly retrieved from the PDB.

### 3.2 Diversity of dimeric, trimeric, tetrameric, and pentameric fragments

We next explored the performance and viability of PDBMine in global PDB searching tasks. Certain amino acid residues, such as Leucine and Alanine, are known to occur in higher relative abundance than others in the proteome (Shen et al., 2006; Nacar, 2023). To further validate PDBMine and investigate relative abundances of amino acids, we conducted a global search of every possible k -mer for k = 1 to 5. For each k , we generated an exhaustive list of all $20^{k}$ possible amino acid sequences of length k . For example, when k = 1, there are 20 possible sequences: the amino acids alone. Since the abundance of individual amino acids is known, the comparison of PDBMine’s results with the known knowledge will serve as a validation. When k = 2, there are 400 possible sequences: Ala-Ala, Ala-Arg, … , Val-Tyr, Val-Val. PDBMine was queried using Method 2 to count the occurrences of each such sequence in the PDB. Following Method 2, the specified window size was always equal to the length of the k -mer (eg. the trimer AAA was queried with a window size of 3), such that PDBMine only generated one window from each k -mer. This experiment not only validates PDBMine, but provides novel insight into the representation of short amino acid subsequences.

In addition to exploring the frequency of individual k -mers, we also investigated the statistical independence of their occurrences. To quantify this, we examined the lift of k -mer occurrences by comparing the observed probability of a k -mer to its expected probability under the assumption that the amino acids occur independently. This metric—referred to as departure from independence, $d_{i}$ —is defined in Equation 1, where $a_{i}$ denotes the i -th amino acid in a sequence of a k -mer, and $P_{\mathrm{obs}}\left(a_{1}\cdots a_{k}\right)$ represents the observed probability of the full k -mer sequence occurring in the PDB. The denominator (Equation 2) is the product of the observed probabilities of each amino acid in the k -mer occurring individually, representing the expected, joint probability of observing the amino acids under an assumption of independence. If the amino acids in a k -mer truly occur independently, then the observed probability of a k -mer will be consistent with the expected probability (i.e., $d_{i}=1$ ). However, this scenario, indicating independence of amino acid occurrences, is not always the case. Deviations from this baseline indicate statistical dependence, meaning that $d_{i}>1$ suggests that the constituent residues co-occur more frequently than expected by chance, while $d_{i}<1$ indicates underrepresentation. This measure enables a quantitative assessment of sequence context effects across the proteome. We hypothesize that longer k -mers will show a greater departure from independence. For example, the dimer Ala-Ala is expected to behave more independently than the tetramer AAAA, which in turn is expected to be more independent than the pentamer AAAAA.

$$d_{i}=\frac{P_{\text{obs}}\left(a_{1}\cdots a_{k}\right)}{P_{\text{joint}}\left(a_{1}\cdots a_{k}\right)}$$ (1)

$$P_{\text{joint}}=\overset{k}{\underset{i=1}{\prod }}P_{\text{obs}}\left(a_{i}\right)$$ (2)

### 3.3 Restricted representation of contextualized sequences

PDBMine offers unique insights into the statistics of dihedral angles for k -mer sequences across the PDB. Given a short amino acid subsequence consisting of a residue of interest (e.g., the center residue) and its surrounding residues, PDBMine can be queried using Method 2 to find full length matches of the subsequence. The distribution of dihedral angles for the residue of interest is contextualized by its neighboring amino acids; this set of context-specific dihedral angle distributions represents a restricted R-space.

We define the target residue as the center residue in the case of an odd-length k -mer, and as the first of the two central residues in the case of even-length k -mer. By focusing on this residue, we can estimate the two-dimensional probability distribution of its ϕ and ψ angles, conditioned on its surrounding residues. This allows us to compare the dihedral angle distributions of the same residue across different contexts. As an example, we estimated the probability distributions of the residue Alanine (A) in the pentamers GLALS and DEAKK, which are subsequences of the protein 6POO (residues 318-322 and 372-376, respectively) (Manne et al., 2020).

Expanding on the idea of contextualized dihedral angle representations, we also examined the effect of increasing window size (i.e., k -mer size) on the dihedral angle distribution of a given residue. The amino acids surrounding residues of interest serve as context, and, as the level of context increases and the sequences become more unique, we expect to see greater restriction on the dihedral angle distributions of residues of interest. To investigate this, we queried PDBMine using Method 2 on four subsequences centered around Lysine (K) at position 75 in the protein 2RJ7: PKVL, QPKVL, QPKVLT, and PQPKVLT (Alfaro et al., 2008). These were selected to evaluate whether longer and more unique amino acid contexts result in more restricted conformational distributions for the same residue.

### 3.4 Applications of PDBMine in structural motif discovery

PDBMine can be used to aid protein structure determination by mining the Protein Data Bank (PDB) for occurring sequence-structure motifs, making it especially useful in fragment-based modeling approaches. It can improve *de novo* structure prediction by providing high-quality, sequence-specific fragment libraries, and support homology modeling by identifying structural motifs that match regions of the target sequence, including poorly conserved loops. In crystallographic model building, PDBMine helps model ambiguous or missing regions by suggesting plausible structural fragments. Additionally, it supports structure-guided mutagenesis and protein design by evaluating the structural viability of new sequence motifs. PDBMine can also enhance machine learning-based structure prediction by contributing structural priors or features derived from known sequence-structure relationships.

In this work, we highlight the use of PDBMine in protein structure validation. The application stems from the concept of restricted representations that have been observed in other structures with a similar subsequence motifs. When queried with longer, more contextualized amino acid sequences, PDBMine identifies the restricted R-space that residues of interest tend to occupy. This concept can be expanded beyond a single residue by examining the dihedral angle distributions of all residues in a subsequence.

When queried, PDBMine reports the set of dihedral angles for a target residue given its neighboring residues and a window size. Equation 3 describes the likelihood of a dihedral angles for residue i (the target residue) given the neighboring residues (denoted by $a_{i}$ ). While, PDBMine provides a complete set of dihedral angles based on the constraint of neighboring residues, it does not consider the structural conformation of the neighboring residues. For example, it may be more likely for residue i to be in α -helical conformation if its neighboring residues are also in α -helical conformation. Equation 4 describes an alternative model of evaluating the likelihood of dihedral angles for residue i considering the neighboring amino acids and their conformational information as joint constraints. In general, each residue can assume one of three conformational states (corresponding α -helical, β -strand, and left-handed-helical regions) (Lovell et al., 2003). It is therefore possible for PDBMine and the formulation stated in Equation 4 to confine the dihedral angles of the target protein to a single structural conformation.

$$P\left(\left(\phi_{i},\psi_{i}\right)|a_{1}\ldots a_{i}\ldots a_{k}\right)$$ (3)

$$P\left(\left(\phi_{i},\psi_{i}\right)|\left\{\left(a_{1},\phi_{1},\psi_{1}\right)\cdots \left(a_{i}\right)\cdots \left(a_{k},\phi_{k},\psi_{k}\right)\right\}\right)$$ (4)

In general, two approaches can be conceived to implement the sequence and structural constraints described in Equation 4. The first approach is to impose the constraints during the course of querying PDBMine’s database. The second approach is to utilize data analytics to organize and the results after querying. Since the first approach is computationally intensive, we adopt the second. More specifically, this approach involves clustering dihedral tuples from each fragment in a high-dimensional space. For example, for a window size of 5, each matching fragment found by PDBMine would contain N=5 pairs of dihedral angles, resulting in 10 total values. In prior methods, these angles are typically analyzed as five independent 2D distributions–one per residue–to study the target residue’s conformational preferences. However, they can also be interpreted jointly as 10-dimensional data points, with each fragment represented as a single vector. Clustering these vectors in high-dimensional space allows us to identify dominant structural motifs associated with the given pentameric sequence.

The hyperdimensional clustering algorithm that we have utilized in this report is summarized in Algorithm 1 below. Continuing the example with the subsequence NKPGD, we: (1) query PDBMine using Method 2 and collect, for example, 2000 full-length fragment matches, each with five dihedral angle pairs, one for each residue in the subsequence. (2) Assemble these results into a list of 2000 10-dimensional data points. (3) Apply Hierarchical Density-Based Spatial Clustering of Applications with Noise (HDBSCAN) (Campello et al., 2015) to find groups of similar points in the 10-dimensional space. Clustering was performed using the angular distance on the ϕ / ψ torus, which treats backbone dihedral angles as periodic coordinates and avoids discontinuities at the ±180° boundaries. We selected HDBSCAN because it does not require prespecification of the number of clusters, identifies noise points, and is well suited for the irregular and multimodal density patterns typical of ϕ / ψ distributions. The final dihedral clusters represent groups of matches (structural motifs) that exhibit similar dihedral angles across the subsequence. The number of clusters found by HDBSCAN is dynamic, and is determined by the density of the points. (4) Identify the medoids of each cluster by computing the pairwise distances among all points in the cluster and selecting the point with the lowest total distance. These medoids are used as representative structural motifs in downstream analysis.

Algorithm 1 **Find full-sequence dihedral angle clusters in the PDB.**

1: Following Method 2, query PDBMine for a given subsequence (length N ) of amino acids. PDBMine finds all full-length matches ( M matches) for the subsequence and returns N pairs of dihedral angles for each match.
2: Assemble the PDBMine results into a list of M high-dimensional data points with dimension 2N .
3: A hierarchical, density-based clustering algorithm—HDBSCAN (Campello et al., 2015) —clusters these points into C (determined dynamically) clusters. Note that the distance metric used for clustering is non-euclidean, since the difference between a dihedral angle of −180 and 180° is 0.
4: The medoids of each cluster are found and serve as representatives. Medoids are the members of each cluster with the lowest total distance to all other members in their clusters.

**FIGURE 2:**
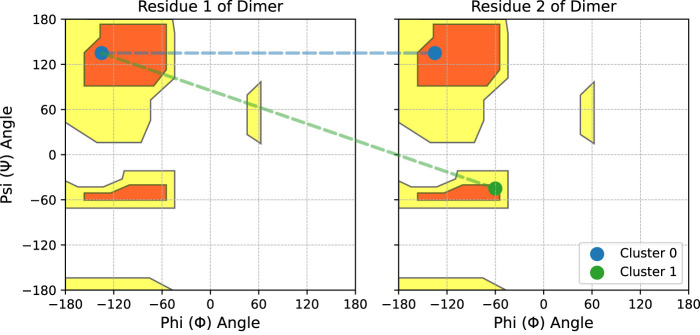
Demonstration of the high dimensional clustering found by Algorithm 1 for some dimer. Scatter points represent clusters found in each 2D distribution independently. In this demonstration, matches for the first residue only form a cluster in the Beta sheet region. Matches for the second residue form clusters in the Beta sheet and Alpha-helical regions. Across both residues, Algorithm 1 will find the two clusters that form in 4D space, as shown by dotted lines.

As illustrated in Figure 2, each cluster identified by Algorithm 1 represents a combination of the residue-level clusters found in the independent 2D distributions. For example, when querying PDBMine with a dimer subsequence (k=2) , we find two independent 2D distributions via previous methods, and a single 4D distribution following Algorithm 1. If each independent distribution forms clusters in only the Beta sheet region of the Ramachandran plot, then the only cluster possible in the 4D distribution is the combination of these two clusters. This cluster is shown in blue in Figure 2. However, if one independent distribution forms a cluster in the Beta sheet region and the other forms a cluster in both the Beta sheet and Alpha helical regions, then the 4D distribution can form two clusters: the combination of the two Beta sheet clusters and the combination of the Beta sheet and Alpha helical cluster. The new cluster in this scenario is shown in green in Figure 2. In general, given 1-3 expected clusters in the independent 2D distributions for each residue in a sequence of length k , there are $1^{k}$ to $3^{k}$ possible clusters to be found by Algorithm 1 in the single high-dimensional distribution.

We hypothesized, however, that the majority of these potential clusters are not exhibited in the PDB—as in, certain combinations of individual clusters in a subsequence do not occur. The clusters that are represented then serve as baselines for what dihedral angles and, in turn, 3D conformations, are possible for different amino acid sequences. To gain insight into the behavior of these clusters, we applied Algorithm 1 to a large sample size of short amino acid sequences. For each k in {4,5,6,7} , 2,700 k -mers were randomly extracted from the amino acid sequences of a set of proteins from PDB (10.8k total sequences). PDBMine was queried for each, and the results were clustered by Algorithm 1. For each k -mer, the number of matches found by PDBMine and the number of clusters found by Algorithm 1 were recorded.

## 4 Discussion

### 4.1 Validation

**FIGURE 3:**
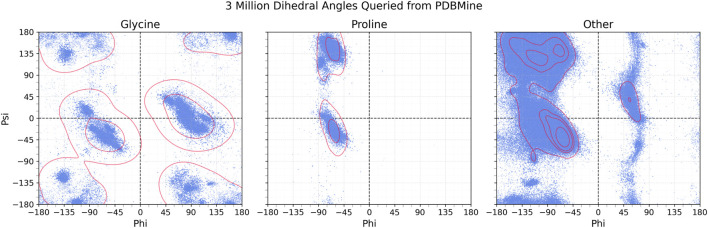
∼ 3 million dihedral angle pairs retrieved from PDBMine using the amino acid sequences of 5 proteins. The angles are split between three plots for Glycine (174k), Proline (150k), and all other residues (2.65M). The Kernel Density Estimation of these distributions are shown in red, outlining dense regions.

The approximately 3 million dihedral angles retrieved from PDBMine are shown in Figure 3. The results are categorized into three groups based on the amino acid: Glycine, Proline, and all other residues. As expected, the distributions adhere to established Ramachandran-space restraints for each group. For the Glycine residues (174k), we observe high-density regions corresponding to the five distinct conformational clusters typically exhibited by this residue (Ho and Brasseur, 2005). Similarly, results for Proline residues (150k) adhere to the expected restriction of ϕ , centered around approximately −65° . Finally, for all other residues (2.65M), the dihedral angles cluster in well known regions of the Ramachandran plot, including alpha-helical, beta sheet, and left-handed-helical regions. With a sample size of approximately 3 million angles and four different window sizes used for querying, these results demonstrate that PDBMine accurately captures conformational trends across diverse sequence contexts. This supports the reliability of PDBMine as a tool for extracting dihedral information from the PDB.

### 4.2 Results of dimer, trimer, and tetrameric fragments

**FIGURE 4:**
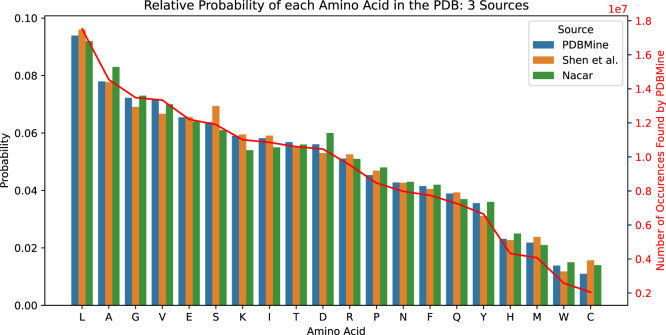
Relative probability of each amino acid found by three methods. In blue are the probabilities in the PDB found by querying PDBMine for each individual amino acid. In orange are the probabilities of amino acids in the SwissProt database found by Shen et al. (Shen et al., 2006). In green are the probabilities in a subset of the PDB found by Nacar (Nacar, 2023). The red line shows the number of occurrences of each amino acid in the PDB, found through PDBMine.

We conducted a global search of all k -mers for k = 1 through k = 5. For each value of k , we generated an exhaustive list of all possible amino acid sequences of that length. For example, at k = 1, there are 20 sequences: the individual amino acids. For k = 2, there are 400 combinations (e.g., Ala-Ala, Ala-Arg, … , Val-Tyr, Val-Val), and so on. At k=1 , PDBMine identified 186,574,282 residues in total in the PDB, with an average of 9.3 ± 4.1 million matches per amino acid. Leucine and Alanine were the most abundant, with about 17.5 and 14.5 million occurrences in the PDB, respectively. Cysteine and Tryptophan are the least common, with 2.0 and 2.6 million occurrences, respectively. For each amino acid $a_{i}$ , we calculated its relative probability as $P_{\text{obs}}\left(a_{i}\right)=N_{i}/M$ , where $N_{i}$ is the number of matches for amino acid $a_{i}$ and M is the total number of amino acids in the PDB. The estimated probability of each amino acid is highly consistent with the results found by Nacar and Shen et al., which performed similar analyses on a subset of the PDB and the SwissProt database, respectively (Shen et al., 2006; Nacar, 2023). The total number of matches and probability for each amino acid found by PDBMine, Shen et al., and Nacar are summarized in Figure 4. These results further validate the accuracy of PDBMine’s results and demonstrate its ability to extract valuable statistics from the PDB.

**FIGURE 5:**
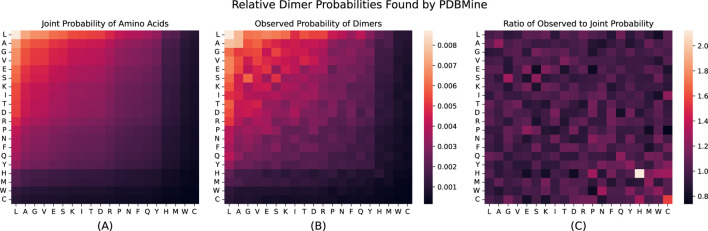
**(A)** Shows the joint probability of Dimers solely based on the observed probability found for each acid acid independently: $P_{\text{joint}}\left(A_{j}A_{i}\right)=P_{\text{obs}}\left(A_{j}\right)P_{\text{obs}}\left(A_{i}\right)$ for all dimer combinations $A_{j}A_{i}$ . **(B)** Shows the observed probability for each dimer found through PDBMine. **(C)** Shows $d_{i}$ : the ratio of observed probability to joint probability for each dimer $\left(P_{\text{obs}}/P_{\text{joint}}\right)$ . For each heatmap, boxes correspond to amino acid $A_{j}A_{i}$ , where $A_{j}$ and $A_{i}$ are the amino acids on the vertical and horizontal axes, respectively. The first two subplots are shown on the same color scale. In all plots, the order of the columns and rows are sorted based on the individual amino acid probabilities.

Next, we examined the results of the global search for the 400 possible dimers (Ala-Ala, Ala-Arg, … , Val-Tyr, Val-Val). Again, the total number of occurrences in the PDB was recorded for each dimer, with an average of 460 ± 297 thousand. As before, the probability of each dimer, $P_{\text{obs}}\left(a_{1}a_{2}\right)$ , was estimated. These probabilities are shown in Figure 5B. We also calculated the joint probability of each dimer $P_{\text{joint}}\left(A_{i}A_{j}\right)=P_{\text{obs}}\left(A_{i}\right)P_{\text{obs}}\left(A_{j}\right)$ , where $A_{i}$ and $A_{j}$ are the amino acids that make up the dimer and $P_{\text{obs}}$ is the observed probability of individual amino acids found previously. These probabilities are shown in Figure 5A, and represent the expected probabilities of each dimer occurring based solely on the independent results for monomers. There is a significant discrepancy between the two sets of probabilities. Figure 5C shows the ratio of $P_{\text{obs}}$ to $P_{\text{joint}}$ for each dimer (the previously defined $d_{i}$ ), highlighting the dimers that appear much more often in the PDB than expected based on their individual abundance $\left(d_{i}\gg 1\right)$ . One striking example is the dimer HH, which appears 2.12 times more than expected under independence. This occurrence cannot be fully explained by the low abundance of Histidine alone–other low abundance residues such as Methionine ( $P_{\text{obs}}\left(M\right)$ = 0.02), do not form dimers that exhibit this behavior. For instance, MM appears only 1.22 times more than expected. This elevated frequency of HH may reflect underlying biological or structural functions. Tandem histidines are often involved in metal coordination and stabilization of enzyme active sites (Amrein et al., 2012; Hegg and Que, 1997). The imidazole side chain of histidine allows for flexible protonation and interaction with metal ions, and the presence of adjacent histidines can enhance local binding affinity or structural specificity (Lu et al., 2001). Thus, the high $d_{i}$ value for HH may not be a statistical artifact, but rather a signature of functional motif enrichment in the proteome. These findings highlight the potential influence of local sequence context and may reflect evolutionary or structural preferences in dimer formation.

In addition to their biological relevance, some of these overrepresented k -mers may reflect procedural artifacts in protein production. For example, the elevated frequency of tandem histidines (HH) is likely influenced by the widespread use of poly-histidine tags in recombinant protein purification protocols (Hochuli et al., 1987). These tags, typically consisting of six or more consecutive histidine residues–are engineered into constructs to facilitate affinity chromatography and are not reflective of native protein sequences. Other outlier sequences may similarly arise from engineered linkers, mutations, or expression system artifacts. These possibilities highlight a broader utility of PDBMine by identifying statistically enriched or absent sequence motifs, the platform can reveal both biologically meaningful patterns and systematic biases in the PDB.

These patterns continue with k = 3, 4, and 5. PDBMine was queried with 8,000 trimers, 160k tetramers, and 3.2 million pentamers, covering all possible amino acid combinations for these sequence lengths. The number of occurrences for each sequence is summarized in Table 1. At this stage, we begin to observe amino acid combinations that never appear in the PDB. While all trimers are present, approximately 1.9% of tetramers and 22% of pentamers yielded no matches through PDBMine. As the window size increases and sequences become more specific, the likelihood of unrepresented combinations rises. For both sequence lengths, the amino acids most likely to be in unrepresented k -mers are Cysteine, Tryptophan, Methionine, and Histidine. This is expected, as these amino acids are among the least frequent, and therefore appear in fewer multi-residue combinations.

**TABLE 1:** Number of occurrences found by PDBMine for each k -mer. The average, minimum, and maximum number of occurrences are shown for each k .

Window size (k)	Number of occurrences		
	Average	Min	Max
1	$9.33\pm 4.10\times 10^{6}$	$2.05\times 10^{6}$	$17.5\times 10^{6}$
2	$4.60\pm 2.97\times 10^{5}$	$2.90\times 10^{4}$	$1.61\times 10^{6}$
3	$2.27\pm 1.95\times 10^{4}$	$1.91\times 10^{2}$	$1.50\times 10^{5}$
4	$1.12\pm 1.34\times 10^{3}$	0.00	$5.96\times 10^{4}$
5	55.4±145	0.00	$3.06\times 10^{4}$

**FIGURE 6:**
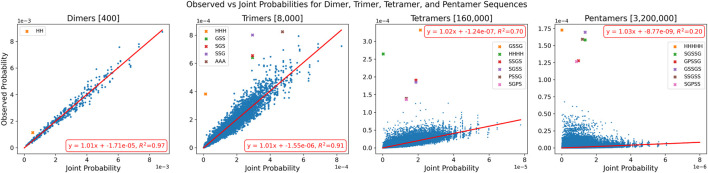
Observed vs. Joint Probabilities of Dimers, Trimers, Tetramers, and Pentamers. On the x-axes are the joint probabilities of each k -mer based on the observed probabilities of individual amino acids. On the y-axes are the observed probabilities of each k -mer found through PDBMine. Outlier sequences are highlighted. Regression lines of best fit and the corresponding equations and $R^{2}$ values are shown in red.

Next, as before, the number of occurrences found in the PDB for each k -mer was used to calculate observed probabilities, and the probabilities of individual amino acids were used to compute the expected joint probabilities. The relationship between these two sets of probabilities, along with the line of best fit is shown in Figure 6 for each value of k . The slope of this line represents the best approximation for the value of $d_{i}$ across different k -mer lengths. As in previous analyses, certain k -mers exhibit $d_{i}\gg 1$ , and are observed at much higher frequencies than expected. These outliers are highlighted in Figure 6. Each regression line for k has a slope of approximately 1 and an intercept near 0, initially suggesting a one-to-one linear relationship between expected and observed probabilities. However, we also observe a sharp decline in the $R^{2}$ value of each regression line as k increases, indicating a deterioration in the dependency between the variables. For dimers and trimers, with $R^{2}$ values of 0.97 and 0.91, respectively, the relationship between expected and observed probabilities is well modelled by a one-to-one regression line. Aside from the highlighted outlier sequences, these dimers and trimers can be reasonably predicted using the individual abundances of their constituent amino acids. However, this trend does not quite hold for the longer tetramers and pentamers sequences, where the predictive power of individual abundances rapidly deteriorates. These results directly support the hypothesis introduced in Section 3.2: as sequence length increases, k -mers exhibit greater departure from statistical independence. The declining $R^{2}$ values confirm that the observed frequencies become increasingly unpredictable from individual amino acid probabilities alone, reflecting the growing context dependence captured by the $d_{i}$ metric. The $R^{2}$ value for tetramers drops to 0.70, indicating a much weaker relationship between expected and observed probabilities at k=4 . For pentamers, the breakdown is even more pronounced with an $R^{2}$ value of 0.20, which suggests that the behavior of amino acids are far from independent in groups of five and the relative abundances of pentamers cannot be meaningfully predicted based on individual amino acid frequencies alone.

The fact that we begin to see unrepresented k -mers for k=4 and a substantial fraction (22%) for k=5 underscores the selective pressures and constraints that govern protein sequence evolution and structure formation. The decreasing $R^{2}$ values in Figure 6 provide a quantitative measure of this increasing context-dependence. For dimers and trimers, the observed frequencies are largely driven by the independent probabilities of the amino acids that make them up. However, for tetramers and especially pentamers, additional factors beyond simple amino acid abundance appear to influence sequence occurrence. These may include stereochemical constraints due to side chain interactions, as well as evolutionary history. The highlighted outlier sequences are particularly interesting. The fact that certain k -mers appear much more frequently than predicted based on their constituent amino acid frequencies suggests strong selective pressures favoring these specific combinations. Further investigation into the structural and functional roles of these enriched sequences could yield insight into protein design principles and evolutionary constraints.

### 4.3 Restricted representation of contextualized sequences

**FIGURE 7:**
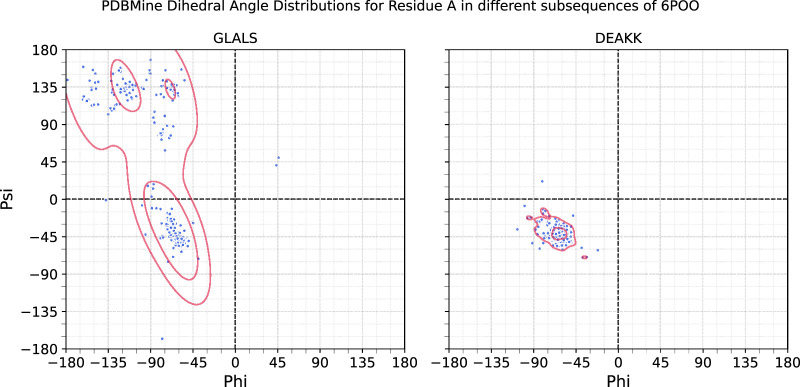
Dihedral angle distributions queried from PDBMine for the residue Alanine occurring in different pentamers from the protein 6POO: GLALS at residues 318-322 and DEAKK at residues 372-376. Overlaid are the Kernel Density Estimations of the dihedral angles for each. Here, the same residue demonstrates different patterns due to the amino acids that neighbor it. Both queries yielded about 380 matches.

To investigate the influence of sequence context on dihedral angle distributions, we examined the residue Alanine (A) in two different pentameric contexts: DEAKK and GLALS. Using Method 2, we retrieved approximately 380 matches for each subsequence. The estimated 2D ( ϕ , ψ ) angle distributions for the central Alanine were compounded using Kernel Density Estimation and are shown in Figure 7. The distributions differ markedly, the Alanine in DEAKK exhibits a concentrated cluster around the alpha-helical region of the Ramachandran plot, whereas the Alanine in GLALS displays a broader distribution spanning both alpha-helical and beta-strand regions. These differences demonstrate that the conformational preferences of a residue are strongly influenced by its immediate sequence context.

**FIGURE 8:**
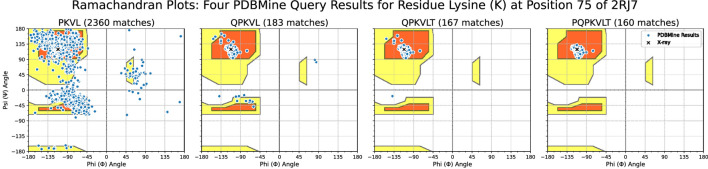
Ramachandran plots of PDBMine results for amino acid subsequences of the protein 2RJ7. Each plot shows the dihedral angles of Lysine (K) found when PDBMine is queried with the overlapping subsequences shown on subplot titles. The chosen subsequences demonstrate the effect of gradually increasing the number of surrounding residues sent to PDBMine. Shown in black X markers are the dihedral angles of the Lysine in the model of the protein found through X-ray crystallography. Shown in blue are the results from PDBMine. The background outlines the typical Ramachandran space of dihedral angles.

We next examined the effect of increasing surrounding context when querying PDBMine for a residue of interest. Figure 8 shows the results of Method 2 queries using four subsequences of increasing length centered on the Lysine at position 75 of the protein 2RJ7. As the sequence length increases, the results become more context-specific and converge toward the dihedral angle values reported from X-ray crystallography. With three residues of context, the subsequence PKVL yields 2,360 matches. The resulting distribution of dihedral angles for the Lysine spans the entire Ramachandran space, indicating that a 4-mer is not specific enough to yield a meaningful conformational distribution. As subsequence length increases, however, the distribution of dihedral angle begins to converge around the beta sheet region. When queried with six residues of context, the subsequence PQPKVLT yields only 160 matches, but these matches cluster tightly around the experimentally determined dihedral angles for Lysine-75 in 2RJ7: ϕ=−112 °and ψ=119 . These 160 matches occur in 146 distinct PDB entries that contain an exact match to the subsequence PQPKVLT, with sequence lengths ranging from 259 to 1,102 residues. The structure of 2RJ7 itself was excluded from the query results to avoid circularity. These results demonstrate how, as the subsequence length of a PDBMine query increases, the results yield more contextualized matches, clustering around particular sections of the R-Space. In this way, PDBMine shows its potential as a tool for structure validation and prediction.

### 4.4 Applications of PDBMine - structure validation results

**FIGURE 9:**
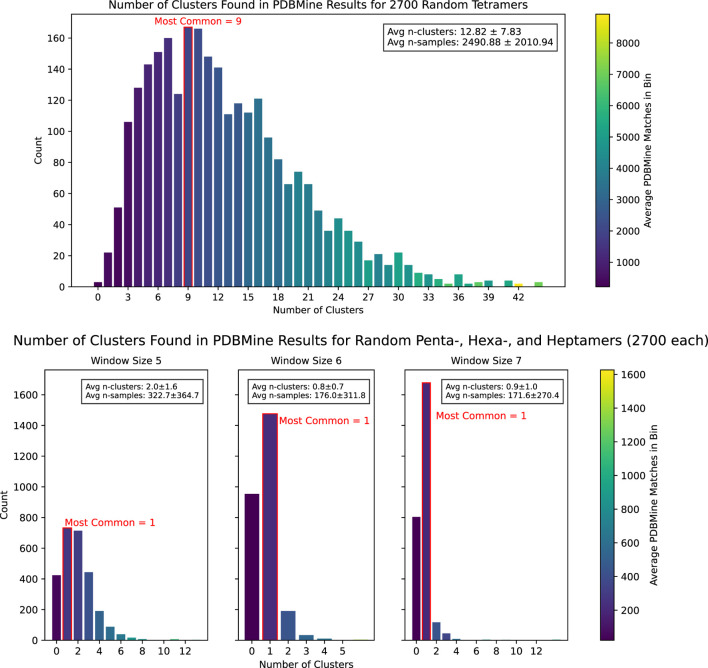
The results of Algorithm 1 on PDBMine queries for 10,800 randomly selected k -mers. For each window size, a histogram shows the distribution of the number of clusters found for each k -mer. The color of each bar represents the average number of matches found by PDBMine for the k -mers that fall into that bin.

To demonstrate PDBMine’s potential to serve as a method of predicted structure validation, we employed Algorithm 1 to conduct a study of 10.8k amino acid sequences of lengths 4, 5, 6, and 7. For each sequence, the number of dihedral conformation clusters exhibited in the PDB was found through PDBMine and Algorithm 1. The results are shown in Figure 9.

Given that each residue in a sequences of length k can form 1-3 independent dihedral clusters, there are theoretically $1^{k}$ to $3^{k}$ possible cluster combinations detectable by Algorithm 1. However, our experiment found that far fewer clusters are actually exhibited in the PDB. As summarized in Table 2, longer window sizes tend to produce fewer clusters: sequences of length six and 7 generally form only one cluster, while sequences of length 5 form two on average. This is consistent with previous results and supports the use of PDBMine as a viable tool for structure validation and prediction.

**TABLE 2:** Possible number of clusters found by PDBMine and Algorithm 1 for amino acid subsequences of different lengths compared to the observed number.

Window size (k)	Possible n-clusters (1 to $3^{k}$ )	Observed n-clusters (mean ± std)
4	1–81	12.84 ± 7.84
5	1–243	2.0 ± 1.6
6	1–729	0.8 ± 0.7
7	1–2,187	0.9 ± 1.0

**FIGURE 10:**
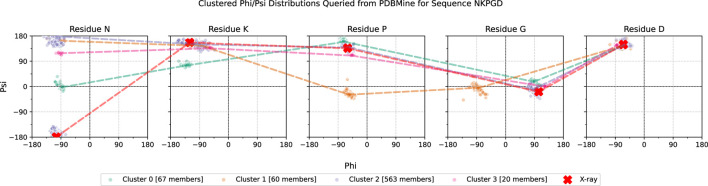
Example of a PDBMine distribution for each residue of a 5-residue window, NKPGD, which occurs at positions 98-102 in the protein 7EIK. Each subplot shows the dihedral angles queried from PDBMine for one residue of the subsequence. The red X marks show the dihedral angles of each residue in the X-ray Crystallography model of 7EIK, with red dotted lines outlining their connection. The remaining dotted lines are representative of four different clusters, found by PDBMine and Algorithm 1.

**FIGURE 11:**
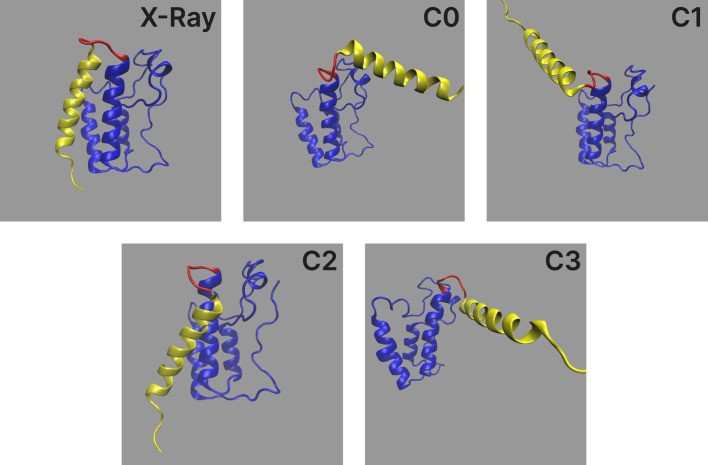
Protein 7EIK with residues 1-97 in blue, 98-102 in red, and 102-125 (end) in yellow. The first model (labelled X-Ray) shows the structure of 7EIK found through X-ray crystallography and retrieved from the PDB. In each of the following molecules, the dihedral angles of residues 98-102 (red) are set to the values found in each cluster shown in Figure 10. Clusters 0 to 3 are shown in order (labelled C0-C3).

An example of PDBMine results clustered by Algorithm 1 is shown in Figure 10, illustrating the implications of these experimental results. While each individual residue-level subplot exhibits 1-3 clusters, not all combinations of these clusters across the full subsequence appear in the PDB, as expected. This provides valuable insight into which conformational formations of a k -mer are structurally feasible and represented. Figure 11 shows the hypothetical conformations of the protein 7EIK for each of these dihedral angle clusters (Zheng et al., 2022). The dihedral angles of the residues in the highlighted coil region are replaced with the angles in each cluster found by Algorithm 1. Each cluster forms a different structure, serving as a basis for the possible formations of this region of the protein. By identifying these experimentally observed conformational clusters, PDBMine can help guide subsequent modeling or refinement steps, particularly when combined with downstream analyses such as molecular dynamics or energy minimization.

One of the most persistent challenges in structure modeling lies in accurately reconstructing loops and flexible regions, where experimental density is often incomplete and conformational heterogeneity is high. The dihedral angle clusters retrieved by PDBMine provide an empirical foundation for constraining such regions during model building. Recent work by Pandala et al. (Pandala et al., 2025) leveraged PDBMine derived torsion angle distributions to evaluate and refine AlphaFold2 models from the CASP14 competition, demonstrating that PDBMine based likelihood analysis can pinpoint residues in flexible or mismodeled regions and guide structural correction. Similarly, in our own analyses, the distinct clusters identified for the NKPGD subsequence define a finite set of backbone conformations that can be mapped onto missing or ambiguous residues. These empirically observed ϕ / ψ combinations serve as physically realistic priors that restrict conformational search space while preserving structural diversity. Together, these findings highlight PDBMine’s potential to bridge experimental structural data with predictive modeling frameworks, enhancing refinement of flexible or poorly resolved regions.

## 5 Conclusion

PDBMine provides a flexible and interpretable platform for analyzing large-scale sequence–structure relationships in the Protein Data Bank. In this work, we demonstrated how PDBMine can be used to explore amino acid sequence frequencies, evaluate the conformational effects of local sequence context, and perform high-dimensional clustering of dihedral angles across protein fragments. These applications highlight how PDBMine supports a range of research needs, from identifying enriched sequence motifs to validating predicted structures and modeling context-specific conformational variability.

Beyond demonstrating platform capabilities, our results reveal several structural principles underlying protein architecture. A key finding is the systematic breakdown of statistical independence in amino acid occurrence as k -mer length increases. While dimers and trimers exhibit high correlation ( $R^{2}\approx 0.97$ and 0.91) between expected and observed frequencies, this correlation deteriorates sharply for tetramers $\left(R^{2}\approx 0.70\right)$ and collapses for pentamers $\left(R^{2}\approx 0.20\right)$ , with many longer k -mers either overrepresented or absent in the PDB. These trends suggest strong local constraints arising from evolutionary pressures, stereochemical compatibility, and structural feasibility. The departure-from-independence metric introduced here provides a quantitative lens for assessing these effects.

Additionally, we demonstrated that the structural preferences of individual residues are highly sensitive to their local context. As subsequence length increases, PDBMine returns more constrained dihedral angle distributions that converge toward experimentally observed geometries. High-dimensional clustering of full-sequence dihedral tuples revealed that, despite a combinatorial explosion in potential conformations (e.g., $3^{5}=243$ for 5-mers), only a small fraction are actually represented in the PDB. These findings support the use of empirically grounded motif constraints for tasks such as loop modeling, fragment assembly, and flexible region refinement.

Together, these results demonstrate the range of structural questions that PDBMine can help address. What we have presented here is just a small sample of what is possible. PDBMine was designed to be general and extensible; the examples in this paper serve as starting points for more detailed applications. Future work could include building machine learning models based on PDBMine-derived features, integrating side-chain geometries such as χ angles, or extending the system to explore noncanonical residues and post-translational modifications. Prior work has shown that χ angle clustering reveals consistent rotameric patterns that complement backbone-based structure prediction (Van Beusekom et al., 2021), and incorporating such analyses into PDBMine represents a promising direction for expanding its structural modeling capabilities. By making structural statistics easily queryable, PDBMine provides a foundation for a wide variety of downstream tools and studies. We hope this work encourages others to explore new directions using PDBMine to better understand the statistical and structural rules that govern protein behavior.

## Data Availability

Publicly available datasets were analyzed in this study. This data can be found here: The original structural data is publicly available from the Protein Data Bank (PDB) at: https://www.rcsb.org. The processed dataset used in this study is not hosted online due to size constraints. However the complete PDBMine source code, Docker configuration, and documentation for setup and usage are publicly available through the Valafar Lab GitHub repository at: https://github.com/ValafarLab/PDBMine. A publicly accessible instance of the current PDBMine backend is also available at: https://ifestos.cse.sc.edu/PDBMine for convenient browser based access. Because this deployment is hosted on departmental infrastructure, long-term availability is not guaranteed; the supported and fully reproducible access methods remain the Docker container and REST API.
